# Simultaneous Discovery of Cell-Free DNA and the Nucleosome Ladder

**DOI:** 10.1534/genetics.118.300775

**Published:** 2018-04-26

**Authors:** Steven Henikoff, George M. Church

**Affiliations:** * Basic Sciences Division, Fred Hutchinson Cancer Research Center, Seattle, Washington 98109; † Howard Hughes Medical Institute, ‎Seattle, Washington 98109,; ‡ Department of Genetics, Harvard Medical School, Boston, Massachusetts 02115

**Keywords:** chromatin, apoptosis, precision medicine, molecular biology

A remarkably prescient paper published by Robert Williamson in 1970 was the first description and correct interpretation of both the apoptotic origin of cell-free DNA and the subunit structure of chromatin. Thus, the original observation that forms the basis for this powerful precision medicine strategy also played a central role in the discovery of the nucleosome.

Many seminal discoveries are overlooked because they are ahead of their time (STENT 1972). Here we present one of these stories: the dual discovery of the origin of cell-free DNA and the subunit structure of chromatin.

cfDNA from human plasma is a noninvasive diagnostic tool that is rapidly becoming a standard in the clinic. Prenatal diagnosis for chromosome abnormalities and some single-gene disorders is now routinely done by assaying fetal cfDNA in the mother’s blood plasma (Bunkar *et al.* 2018). After organ transplantation, cfDNA from the donor allograft released into the plasma can serve as a sentinel of rejection (Snyder *et al.* 2011; Daly 2015; Bloom *et al.* 2017; Schütz *et al.* 2017). Apoptosis within a tumor results in the release of sufficient cfDNA into the plasma or cerebral spinal fluid to monitor the course of treatment (Diaz and Bardelli 2014; Cohen *et al.* 2018). The continuing reduction in the cost of DNA sequencing is bringing cfDNA assays into the clinic for the diagnosis of cancer and many other diseases. Thus, cfDNA assays are key drivers of the current personalized medicine revolution.

Although the detection of cfDNA in plasma was first described two decades ago (Lo *et al.* 1997), its generation in dying cells was observed much earlier. In 1970, Robert Williamson described what is now familiar as the “nucleosome ladder” in the cytoplasmic fraction of embryonic mouse liver (Williamson 1970). This dual discovery, which presaged the development of two different research fields, was motivated by Williamson’s attempts to isolate globin mRNA from reticulocytes, where he was stymied by contamination with cytoplasmic DNA fragments. His curiosity about the source of cytoplasmic DNA contamination, and about various suggestions that cytoplasmic DNA might be metabolically active and “functional” akin to mRNA, led him to a systematic study of cytoplasmic DNA. His protocol was simple: excision of embryonic mouse livers, culturing in [^32^P]phosphate- or [^3^H]thymidine-containing medium, lysis and centrifugation to separate nuclei from cytoplasm, and extraction of DNA from the supernatant and pellet. He then characterized the labeled DNA from the cytoplasmic and nuclear fractions by buoyant density centrifugation, polyacrylamide gel electrophoresis (PAGE), DNA depurination fingerprinting, and base composition analysis. In this way, he could compare the physical, informational, and metabolic properties of cytoplasmic DNA and nuclear DNA.

**Figure 1:**
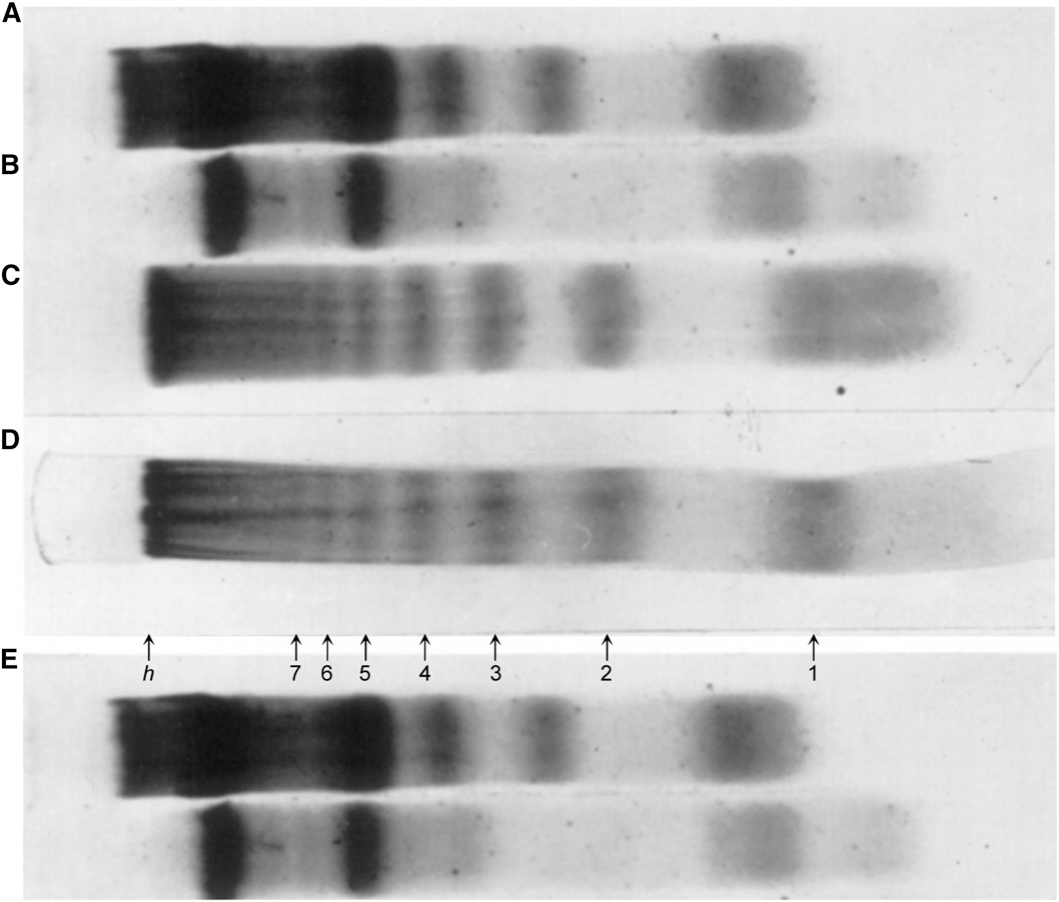
PAGE analysis (2.4%) of DNA fragments from embryonic mouse liver. (A) Total cytoplasmic nucleic acid; (B) same after treatment with DNase; (C) same after treatment with RNase; (D) purified cytoplasmic DNA (after RNase treatment of total cytoplasmic nucleic acid and passage through Biogel P30 column; and (E) 141/2-day nuclear DNA. Reproduced with permission from Williamson (1970).

Williamson’s thorough characterization of cytoplasmic DNA using tools available at the time showed that the cytoplasmic DNA was similar to nuclear DNA in buoyant density and base composition, ruling out mitochondrial contamination, and complex fingerprints indicated that it was composed of many different sequences. However, unlike most nuclear DNA, cytoplasmic DNA showed a remarkable electrophoretic pattern, a regular ladder of up to 10 bands (Figure 1). This ladder was sensitive to DNase (Figure 1, A and B), resistant to RNase (Figure 1, C and D), and detectable as a minor component of nuclear DNA (Figure 1E). The electrophoretic mobility of the bands fell on a straight line on a log Mw *vs.* distance plot, revealing that the fragments corresponded to a series of multiples of a unit length. His estimated Mw = 135,000 for the difference between bands corresponds to ∼200 bp of DNA, the repeat unit that later work showed is the nucleosome repeat length. This left the question: what was its origin?

Because cytoplasmic DNA accounted for as much as 20% of the total DNA in his cultures, Williamson concluded that it was unlikely to consist of replication intermediates. Autoradiography of embryonic liver labeled for 16 hr showed that whereas 30% of the cells were labeled in the nucleus, only 15% of the cells also displayed cytoplasmic labeling, which suggested to Williamson that this subset of cells comprised a distinct class of metabolically active cells. Noting that 14 and 1/2 day-old embryonic liver consists mostly of erythroid cells at several stages of development, he proposed that DNA fragmentation is associated with disintegration of nuclei in dying cells. Although this was 2 years before the term apoptosis was coined (Kerr *et al.* 1972), and long before apoptotic pathways were delineated, Williamson’s interpretation of the cytoplasmic DNA as deriving from nuclear disintegration during cell death is now considered to be correct. For example, cfDNA from plasma shows the same nucleosome ladder as Williamson observed, and analysis of internucleosomal distances (Snyder *et al.* 2016) and subnucleosomal intermediates (Ramachandran *et al.* 2017) as DNA proxies for gene expression confirms the hematopoietic origin of the apoptotic cells. The simultaneous discovery of the nucleosome ladder and the origin of cfDNA in 1970 was thus correctly interpreted by Williamson, respectively 3 years and nearly 3 decades before the biological significance of nucleosomes and the clinical utility of cfDNA were appreciated.

Williamson’s dual discovery was published in the *Journal of Molecular Biology*, the premier molecular biology journal of the day; however, it has remained surprisingly underappreciated relative to subsequent papers that built upon its findings. As described by Kornberg and Klug in a *Scientific American* article in 1981 (Kornberg and Klug 1981), Williamson’s discovery of the nucleosome ladder was a key insight that led to the nucleosome model: “…he had reported sizes for the DNA fragments that were multiples of ∼200 nucleotide pairs. The agreement with the size of fragments expected from the beads-on-a-string idea was remarkably good. From this moment on it seemed the idea must be right.” Yet, the discovery of the nucleosome ladder is widely attributed to a brief 1973 report of the ladder resulting from the *in situ* action of an endogenous nuclease by Hewish and Burgoyne (1973), despite the fact that they did not report the repeat length. In a 1982 interview, Hewish gave credit to Williamson: “…we were influenced by a previous publication of Williamson, who had described a regular series of size classes among DNA fragments in the cytoplasm of cultured cells” (Hewish 1982).

Although Williamson’s seminal findings were eventually recognized in the apoptosis field (Duke *et al.* 1983), his 1970 paper is cited only 1/10th as frequently as Hewish and Burgoyne’s, and almost never for its discovery of the nucleosome ladder. The reasons for the paper’s relative obscurity are unclear. In *Understanding DNA: The Molecule and How it Works*, Calladine *et al.* (2004) asserted that “he thought that these fragments might come from the incomplete synthesis of long DNA molecules, as shown by previous work, rather than from the degradation of intact chromosomes. Because of this his work attracted little attention.” However, this interpretation was one of many that Williamson considered but judged to be unlikely. Leaving no doubt as to what he thought, Williamson’s abstract closed with the sentence: “These findings are consistent with a hypothesis that in this system the DNA isolated from the cytoplasm is a degradation product of nuclear DNA.” Perhaps a more plausible reason for the paper’s obscurity in the chromatin field was an influential *Nature* paper entitled “Subunit structure of chromatin” (Noll 1974), which demonstrated the 200-bp repeat unit of the ladder, but cited Hewish and Burgoyne’s 1973 paper as prior work, and later authors followed suit.

Although Williamson maintained a thriving laboratory subsequent to publication of his 1970 paper, he never followed up on its findings. He has attributed his own lack of interest in the DNA ladder to its being a “structural,” not a “functional,” feature of DNA, and has credited those who realized its significance for developing the nucleosome model that has had such a huge impact in understanding eukaryotic biology. While his modesty is perhaps justified with respect to the part his discovery played in developing the nucleosome model, he deserves full credit for the correctness of his interpretation of what we now know to be the apoptotic origin of cfDNA. As the clinical importance of cfDNA becomes increasingly manifest, Williamson’s pioneering insights from nearly 50 years ago take on increasing significance and merit growing admiration.
